# Oxygen Partial Pressure as a Kinetic Descriptor for Single‐Crystal Morphology in Mid‐Nickel Layered Cathodes

**DOI:** 10.1002/advs.78080

**Published:** 2026-09-29

**Authors:** Hyeonho Park, Sushil Kumar, Sangbeom Kim, Ho Choi, Youngmin Jeon, Taeju Bak, Jisook Hong, M. P. Gururajan, Youngjin Kim, Kyoungdoc Kim

**Affiliations:** ^1^ Graduate Institute of Ferrous and Eco Materials Technology Pohang University of Science and Technology Pohang Republic of Korea; ^2^ Department of Battery Convergence Engineering Kangwon National University Chuncheon Republic of Korea; ^3^ AI & Robotics Convergence Lab POSCO N.EX.T Hub Seoul Republic of Korea; ^4^ Department of Metallurgical Engineering and Materials Science IIT Bombay Powai Mumbai Maharashtra India

**Keywords:** battery, NCM cathode, oxygen atmosphere, phase‐field modeling, sintering

## Abstract

Establishing the single‐crystal morphology essential for durable mid‐nickel layered cathodes requires high‐temperature calcination, in which strongly coupled densification and grain‐growth kinetics constrain scalable processing. Here, we combine controlled‐atmosphere calcination experiments with phase‐field modeling to resolve how oxygen partial pressure regulates the competing kinetics of densification and grain growth. We find that diffusional transport governs early densification, whereas grain‐boundary mobility dominates grain growth. This indicates that processing conditions must be carefully optimized to balance accelerated grain growth with the efficient removal of trapped pores. Increased oxygen partial pressure raises the activation barrier for grain‐boundary migration, suppressing excessive grain growth while maintaining microstructural uniformity, consistent with experimental observations. Based on these findings, we propose an air–oxygen sequential calcination process that dynamically modulates grain‐boundary mobility during the dwell stage, overcoming the trade‐off inherent to single‐atmosphere calcination and enabling time‐efficient single‐crystal formation with enhanced cycling stability. The optimized air–oxygen sequence achieves a capacity retention of 83.04% after 100 cycles at 45°C, compared to approximately 77% under single‐atmosphere conditions. These results establish oxygen partial pressure as an accessible process variable and quantitative kinetic descriptor for predictive single‐crystal cathode synthesis.

## Graphical abstract

Scalable synthesis of single‐crystal mid‐nickel cathodes demands precise kinetic control over competing densification and grain growth. Oxygen partial pressure emerges as a quantitative descriptor of effective grain‐boundary mobility, acting through changes in the apparent sintering activation barrier. A stage‐specific air–oxygen calcination sequence leverages this tunability to achieve time‐efficient single‐crystal formation and improved cycling stability beyond single‐atmosphere processing.

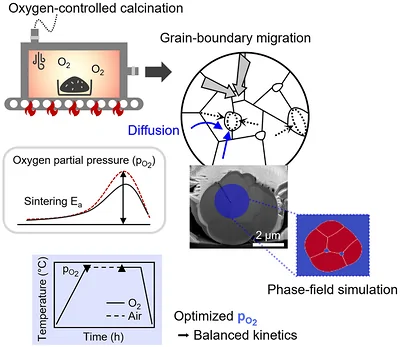

## 1 Introduction

The transition toward high‐voltage and solid‐state battery architectures imposes durability requirements that exceed the capabilities of conventional polycrystalline cathode materials. Single‐crystal (SC) layered oxide cathodes address this demand through enhanced mechanical robustness, suppressed intergranular cracking, and improved electrochemical durability under cycling [1, 2, 3, 4]. Translating these advantages to industrial scale, however, remains constrained by the strongly coupled kinetics of high‐temperature calcination, in which densification, grain growth, and phase transformation proceed concurrently within a narrow processing window [5, 6]. The target SC morphology requires a delicate balance between these competing processes, where modest deviations in temperature, dwell time, or atmosphere shift the microstructure toward incompletely sintered or coarsened particles with trapped internal pores [7, 8]. In the absence of a quantitative kinetic framework linking process variables to microstructural outcomes, optimization remains empirical, and predictive process design remains an open challenge.

Among the process variables conventionally invoked for empirical optimization, temperature most directly governs calcination kinetics by controlling diffusion rates and phase transformation pathways [9, 10, 11]. High‐temperature, short‐dwell protocols accelerate densification but operate within narrow industrial‐scale processing windows, where modest temperature fluctuations promote lithium loss, Li/Ni mixing, and abnormal grain growth [12, 13, 14]. More fundamentally, divergent microstructures observed under nominally identical thermal profiles indicate that kinetic competition between densification and grain growth depends on variables beyond temperature, including atmosphere‐ and interface‐mediated factors that lie outside the scope of thermal control [8, 15]. What remains absent is a process variable that adjusts microstructural kinetics directly without requiring extreme thermal conditions.

Atmosphere control provides such a process variable, influencing both calcination kinetics and phase stability [16, 17]. In high‐nickel chemistries, oxygen‐rich processing has been studied for stabilizing the layered phase against rock‐salt reconstruction and suppressing Li/Ni intermixing [18]. These structural functions combine atmosphere‐induced phase stabilization with kinetic adjustment, with the kinetic role difficult to separate when phase stability is itself oxygen‐sensitive. A quantitative understanding of how atmosphere modulates calcination kinetics therefore requires cathode chemistries where phase stability is largely insensitive to atmosphere, allowing the kinetic role of oxygen to be examined in isolation. Mid‐nickel chemistries (LiNi*_x_* Co*_y_* Mn_1‐_ *_x_*_‐_ *_y_* O_2_ with 0.5 ≤ *x* ≤ 0.7 conventional usage) provide such a regime [19]. At Ni content around 60%, they sit near the air‐to‐oxygen transition in industrial calcination practice and retain phase stability under both atmospheres [20, 21, 22, 23]. A quantitative description of how oxygen restructures the competition between densification and grain growth, however, has remained absent, since experimental observation alone provides limited resolution among the contributions of diffusivity, grain‐boundary mobility, and interfacial energy that govern this competition.

Phase‐field modeling is an established technique for understanding microstructural and morphological evolution during sintering in ceramic systems [24, 25, 26, 27]; however, its application to layered cathode calcination is still emerging. Existing studies have considered the influence of the calcination atmosphere on microstructural evolution during synthesis [28, 29, 30, 31]. These frameworks employ modified Cahn–Hilliard and Allen–Cahn equations to simultaneously describe densification and grain growth [32, 33, 34], while oxygen and lithium transport are modeled using the Cahn–Hilliard equation for O_2_ and Fick's second law for Li, as shown in Equations (1)–(4).

(1) $$\frac{\partial \rho }{\partial t}=\vec{\nabla }\;\cdot \left(D\vec{\nabla }\frac{\delta F}{\delta \rho }-\rho {\vec{v}}_{\mathrm{adv}}\right)$$

(2) $$\frac{\partial \eta_{\alpha }}{\partial t}=-L\frac{\delta F}{\delta \eta_{\alpha }}-\vec{\nabla }\cdot \left[\eta_{\alpha }{\vec{v}}_{\mathrm{adv}}\right]$$

(3) $$\frac{\partial {\tilde{c}}_{O_{2}}}{\partial t}=\vec{\nabla }\;\cdot \left(D_{O_{2}}\vec{\nabla }\mu_{O_{2}}\right)-\vec{\nabla }\cdot \left({\tilde{c}}_{O_{2}}\vec{v}\right)+R_{O_{2}}$$

(4) $$\frac{\partial {\tilde{c}}_{\mathrm{Li}}}{\partial t}=\vec{\nabla }\;\cdot \left(D_{\mathrm{Li}}\vec{\nabla }{\tilde{c}}_{\mathrm{Li}}\right)-\vec{\nabla }\cdot \left({\tilde{c}}_{\mathrm{Li}}\vec{v}\right)+R_{\mathrm{Li}}$$

The model incorporates the dependence of activation energy on local oxygen and lithium concentrations, making it effective for understanding morphological evolution under controlled atmospheres. The detailed formulation is provided in the Supporting Information, and the input parameters adopted from previous studies are summarized in Table S1, where they are treated as effective mesoscale descriptors rather than composition‐specific constants. Despite these advances, atmosphere‐dependent kinetics and realistic precursor geometries have not been jointly incorporated, leaving the role of oxygen partial pressure in microstructural evolution insufficiently resolved.

The present study addresses this gap by combining controlled‐atmosphere calcination experiments with a phase‐field framework that captures oxygen‐dependent microstructural evolution in single‐crystal LiNi_0.6_Co_0.1_Mn_0.3_O_2_ (NCM613), a representative mid‐nickel cathode composition. In the classical description of solid‐state sintering, the competition between densification and grain growth is governed by three classes of quantities: diffusional mass transport, interfacial migration kinetics, and the balance of interfacial driving forces [7]. Together, these determine whether pores are eliminated or become enclosed as grains coarsen, and they are therefore adopted as the candidate kinetic descriptors examined here. The model investigates the influence of key kinetic parameters, including effective diffusivity (*D*), grain‐boundary mobility (*L*), and the interfacial energy ratio (γ_surf_/γ_gb_). The diffusional contribution is further decomposed into pathway‐specific terms, including bulk (*D*_v_), surface (*D*_surf_), and grain‐boundary diffusivities (*D*_gb_). Image‐informed comparisons between FIB‐initialized simulations and experiments corroborate the oxygen‐dependent kinetic description under realistic microstructural conditions. Guided by this experimental–computational analysis, we identify kinetic descriptors for quantitative process design and show that stage‐specific oxygen control, by sequentially modulating grain‐boundary mobility across the calcination dwell, delivers time‐efficient formation of uniform SC mid‐nickel cathodes with improved cycling stability.

## 2 Results and Discussion

### 2.1 Atmosphere‐Dependent Microstructural Evolution

Microstructural evolution during calcination was tracked under two atmospheres, oxygen (p(O_2_) = 0.1 MPa) and air (p(O_2_) = 0.02 MPa). NCM613 secondary particles (∼2–4 µm), initially porous aggregates of primary particles (Figure S1), were heated to 950°C under oxygen (Figure 1a), after which the atmosphere was either kept under oxygen or switched to air. SEM imaging shows that primary‐particle coalescence proceeds more slowly under oxygen than under air (Figure 1b). As shown in Figure 1c, the initial thickness distribution is narrow and centered at ∼250 nm. At 6 h, the mean shifts to ∼540 nm under oxygen with limited broadening, whereas in the air‐switched samples it shifts to ∼1200 nm with substantial broadening and a long tail at larger thicknesses. The two atmospheres thus produce distinct coarsening behaviors in both mean size and distribution width. The (003)/(104) integrated‐intensity ratio, an indicator of layered ordering in which higher values reflect reduced Ni/Li intermixing [18, 35], increases from 1.06 at 0 h to 1.16 at 6 h under oxygen and to 1.17 in the air‐switched samples (Figure 1d). Rietveld refinement of the Ni occupancy on the Li layer, a quantitative measure of cation disorder [9, 11, 12], shows a consistent trend, with values decreasing from 5.94% at 0 h to 5.37% and 4.76% at 2 and 6 h under oxygen, and to 5.22% and 4.51% over the same intervals in the air‐switched samples (Figure 1e and Figure S2). The two atmospheres differ by 0.01 in the (003)/(104) ratio and 0.25% in Ni occupancy at 6 h, both of which may fall within experimental uncertainty. These bulk differences contrast with the more than twofold difference in mean primary‐particle thickness, with ∼540 nm under oxygen and ∼1200 nm in the air‐switched samples. These minimal differences in bulk crystallographic indicators are consistent with the methodological premise that mid‐nickel chemistries near 60% Ni content retain phase stability under both air‐ and oxygen‐processed conditions, allowing the observed microstructural divergence to be attributed predominantly to atmosphere‐driven kinetic effects rather than to phase‐stabilization mechanisms. As shown in Figure 1f, elemental mapping confirms a uniform NCM613 composition within particles without detectable segregation, indicating that particle‐scale composition redistribution does not drive the observed microstructural divergence. The same uniformity is observed for the additional representative calcination conditions (Figure S3). Since energy‐dispersive spectroscopy cannot quantify lithium, the bulk lithium content was additionally determined by ICP‐OES. All samples remain at or above the stoichiometric lithium content, with only a small difference between atmospheres (Table S2), indicating that the atmosphere program does not introduce a meaningful compositional variable. Overall, these results identify oxygen partial pressure as a key regulator of microstructural evolution during NCM613 calcination, while bulk phase evolution and composition redistribution play a subordinate role. The microstructural divergence between the two atmospheres is therefore consistent with origin in the kinetics of grain‐boundary migration and mass transport, which cannot be quantified from ex situ characterization alone and motivates the mesoscale phase‐field analysis that follows.

**FIGURE 1:**
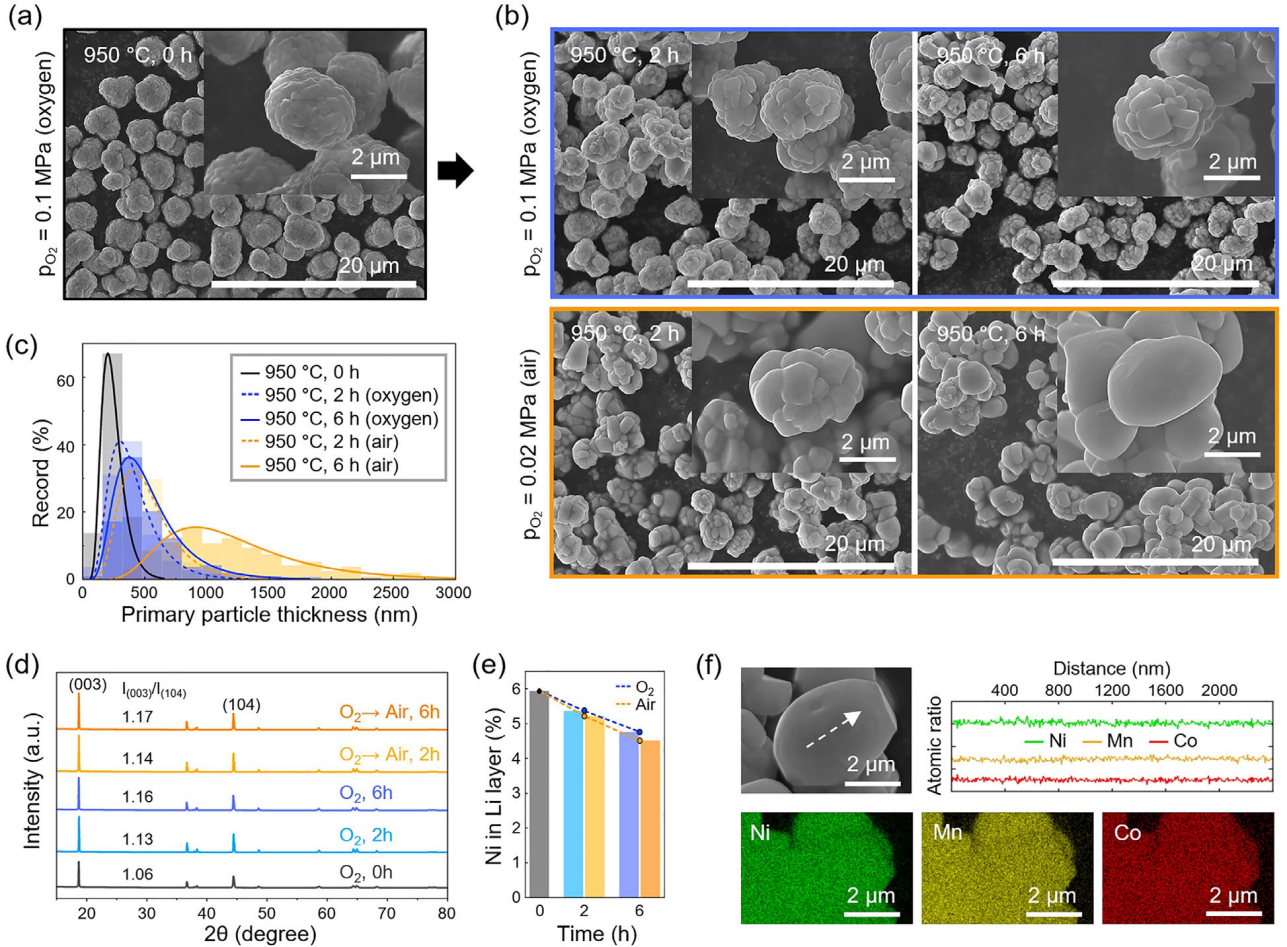
Atmosphere‐dependent microstructural characteristics of NCM613 during calcination. (a) SEM images of secondary particles at 950°C. (b) SEM images after 2 and 6 h under different atmospheres. (c) Primary particle thickness distributions. (d) Ex situ XRD patterns. (e) Cation disorder from Rietveld refinement. (f) Elemental mapping and line profiles of Ni, Mn, and Co for the sample calcined under air for 6 h.

### 2.2 Kinetic Descriptors Governing Densification and Grain Growth

Before examining the effects of oxygen partial pressure on sintering microstructure evolution, we first investigated the fundamental mechanisms governing densification and grain growth during the polycrystalline to single‐crystal transition based on the intrinsic thermodynamic and kinetic properties of NCM613. To isolate the contributions of individual kinetic parameters from initial‐geometry effects, the parametric study was initialized with an idealized 23‐particle configuration. *L*, *D*_surf_, and *D*_gb_ were varied by one to two orders of magnitude (0.1× and 0.01×), while γ_surf_/γ_gb_ was varied by one order of magnitude; each parameter was varied individually with the others fixed.

Figure 2 illustrates how each parameter influences microstructural evolution and the resulting morphological changes. The baseline case (Figure 2a) shows progressive inter‐particle coalescence and pore elimination, culminating in a near‐single‐crystal morphology by τ = 18 000. Reducing grain‐boundary mobility (Figure 2b) yields dense but persistently polycrystalline particles, indicating that *L* is the primary driver of grain coalescence. Reducing surface diffusivity (Figure 2c) accelerates densification relative to the baseline, which is attributed to the effective suppression of grain‐boundary migration: delayed surface transport limits the rapid coalescence that would otherwise isolate pores, thereby allowing pore elimination to proceed ahead of grain merging. Reducing grain‐boundary diffusivity (Figure 2d) exerts a comparatively modest effect on both densification and the overall morphological trajectory. Reducing the interfacial energy ratio γ_surf_/γ_gb_ (Figure 2e) alters the thermodynamic balance between surface and grain‐boundary driving forces but has a weaker influence on the kinetic pathway than does grain‐boundary mobility. Together, these results establish that high grain‐boundary mobility promotes rapid grain merging that can trap pores within the interior, while low mobility preserves a dense but polycrystalline state. At short simulation times (τ ≤ 1000), the simulations reproduce the characteristic features of diffusion‐controlled sintering: inter‐particle neck formation, progressive pore shrinkage, and densification preceding any appreciable grain‐boundary migration (Figure S4). This early‐stage behavior is consistent with the theoretical framework of solid‐state sintering [7], providing a physical basis for interpreting the parameter‐dependent morphological evolution at later simulation times.

**FIGURE 2:**
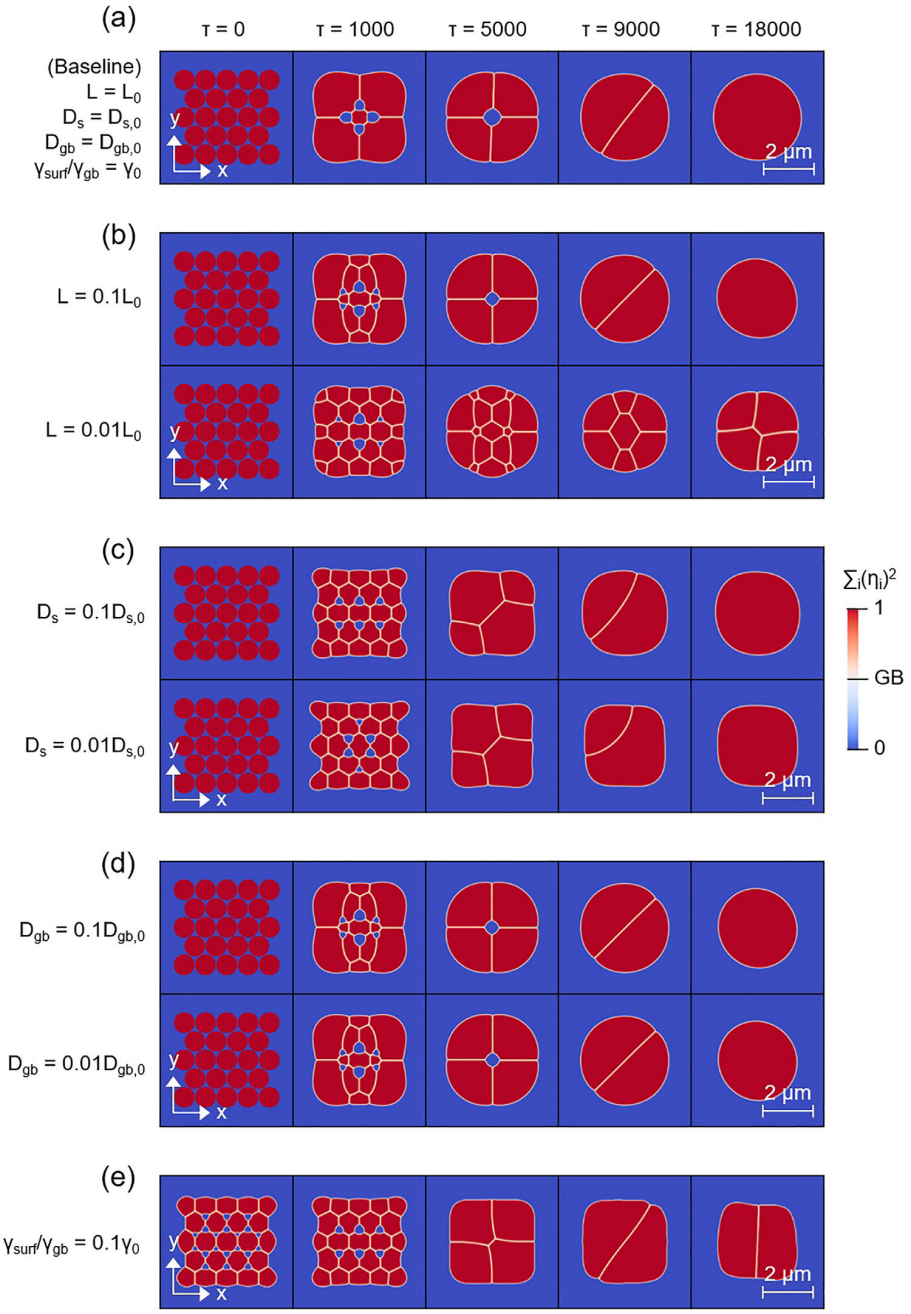
Phase‐field simulations of microstructural evolution under idealized configurations. Simulated microstructural evolution at simulation times τ = 0, 1000, 5000, 9000, and 18000 for (a) the baseline parameter set (*L* = *L*_0_, *D*_s_ = *D*_s,0_, *D*_gb_ = *D*_gb,0_, γ_surf_/γ_gb_ = γ_0_), and with individual reductions of (b) grain‐boundary mobility (*L*), (c) surface diffusivity (*D*_s_), and (d) grain‐boundary diffusivity (*D*_gb_) to 0.1× and 0.01× of their baseline values, and (e) interfacial energy ratio (γ_surf_/γ_gb_) to 0.1× of its baseline value.

Morphological evolution under different parameter values was evaluated relative to the reference case and quantified using two metrics: relative density and area‐equivalent primary particle diameter. Figure 3a shows the temporal evolution of relative density for the 0.01× kinetic parameter conditions and the 0.1× γ_surf_/γ_gb_ condition, selected to highlight the parameter‐dependent trends. At reduced surface diffusivity and mobility, full densification is achieved at τ = ∼4000 and ∼3000, respectively, whereas at reference values it is delayed to τ = 8436 because accelerated grain coalescence isolates pores. This non‐monotonic behavior indicates that grain coalescence outpaces pore elimination, causing pore–grain‐boundary disconnection and delaying pore removal. Figure 3b shows that grain growth is more sensitive to grain‐boundary mobility than to surface or grain‐boundary diffusion. Reducing surface diffusivity delays the single‐crystal transition from τ = 9454 to τ = 11778, while reduced mobility suppresses grain coalescence and stabilizes the polycrystalline state. Diffusional transport dominates early densification, whereas interfacial migration and microstructural topology govern later‐stage evolution [7, 36, 37]. Once pores become disconnected from grain boundaries, further densification is limited and grain‐boundary migration controls subsequent microstructural evolution [38, 39]. This behavior is consistent with hollow structures containing large central pores formed under rapid heating in air (Figure S5), where enhanced grain‐boundary mobility likely facilitates pore isolation [39]. Higher initial pore fraction yields greater internal pore retention (Figure S6), further supporting the role of early pore isolation in shaping the final morphology. During continued calcination, these transient hollow structures dissolve as the single‐crystal morphology develops, reflecting a shift from diffusion‐controlled pore elimination to grain‐boundary migration–driven coalescence. To summarize, diffusional transport governs early densification, whereas grain‐boundary mobility dominates grain growth. This suggests that careful optimization of processing conditions is necessary to reconcile rapid grain growth with the effective escape of trapped pores.

**FIGURE 3:**
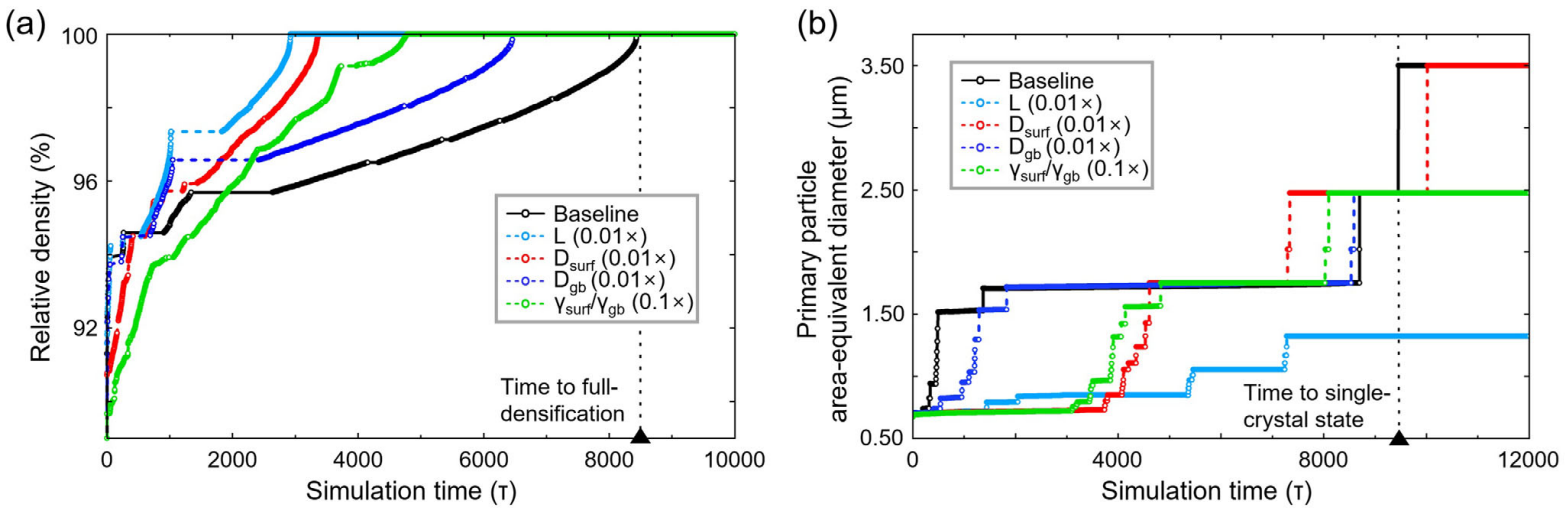
Effects of kinetic parameters on densification and primary‐particle growth obtained from phase‐field simulations. (a) Relative density evolution for the baseline condition and individually reduced kinetic parameters. (b) Area‐equivalent primary particle diameter evolution.

### 2.3 Effect of Oxygen Partial Pressure on Microstructure Evolution

Prior studies on high‐nickel NCM cathodes have shown that elevated oxygen partial pressure increases surface oxygen coverage, raising the activation energy for sintering and suppressing particle growth [16, 28]. Building on this mechanistic understanding, the kinetic effect of oxygen is incorporated into the phase‐field model through the composition‐dependent activation energy (*E*_a_) and diffusivity pre‐exponential factor (*D*_0_) as shown in Equations (5) and (6). The oxygen partial pressure sets the equilibrium oxygen concentration within the particle through the defect‐equilibrium relation (Equation S34). Higher oxygen concentration increases the activation barrier for mass transport, reducing both *D* and *L* and thereby suppressing densification and grain growth [16, 28]. Since grain‐boundary mobility was identified above as the parameter dominating grain coalescence, its suppression is the principal kinetic consequence of increasing the oxygen partial pressure.

(5) $$E_{a}=E_{a,0}+\left(c_{O_{2}}/c_{O_{2},\max}\right)\cdot E_{a,O_{2}}+\left(c_{\mathrm{Li}}/c_{\mathrm{Li},\max}\right)\cdot E_{a,\mathrm{Li}}$$

(6) $$D_{0}=D_{0,0}\;\cdot D_{0,O_{2}}^{\left(c_{O_{2}}/c_{O_{2},\max}\right)}\cdot D_{0,\mathrm{Li}}^{\left(c_{\mathrm{Li}}/c_{\mathrm{Li},\max}\right)}$$

Figure 4a schematically illustrates this mechanism: elevated oxygen partial pressure raises *E*_a_, reducing *L* and slowing interfacial migration, while the representative simulation microstructure at τ = 3500 confirm that grain coarsening proceeds more slowly under O_2_ relative to air. Figure 4b shows that the activation energy under O_2_ (0.1 MPa) increases more steeply and reaches higher values throughout the calcination dwell compared with air (0.02 MPa), consistent with the progressive increase in local oxygen concentration within the NCM particle. The corresponding evolution of grain‐boundary mobility *L* in Figure 4c shows a more pronounced and sustained suppression under oxygen‐rich conditions, consistent with the higher activation barrier. This dependence is not limited to the two atmospheres compared here. Across oxygen partial pressures from 0.02 to 0.10 MPa at 950°C, the activation energy increases monotonically while the effective grain‐boundary mobility decreases correspondingly (Figure S7).

**FIGURE 4:**
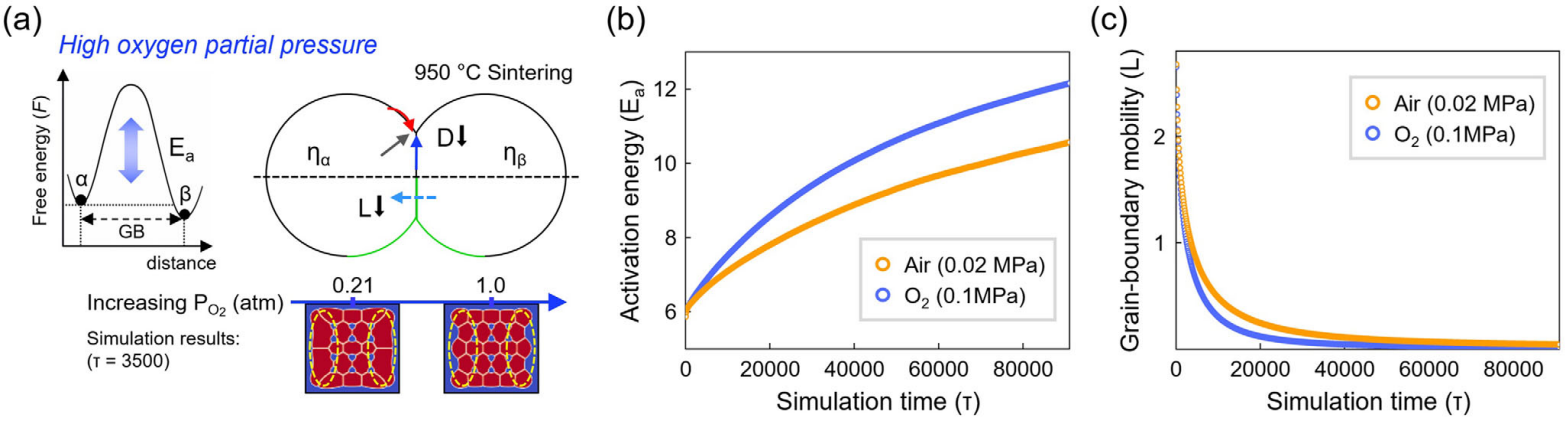
(a) Modeling framework and representative simulations under different oxygen conditions. Temporal evolution of (b) activation energy and (c) grain‐boundary mobility under different oxygen partial‐pressure conditions during calcination.

### 2.4 Experimental–Computational Validation of Oxygen‐Controlled Kinetics

Calcination experiments were performed under different oxygen atmospheres, with O_2_ partial pressures of 0.02 MPa and 0.1 MPa, as schematically illustrated in Figure 5a. During calcination, oxygen and lithium diffuse into the precursor as layered NCM613 forms and the microstructure densifies. Corresponding phase‐field simulations incorporated oxygen and lithium effects through concentration‐dependent activation energies and diffusivity pre‐exponential factors. The initial microstructure was constructed from grain‐orientation fields obtained by FIB ion‐channeling contrast (Figure 5b), with a primary particle size of 0.34 µm. Figure 5c compares simulated and experimental primary particle size evolution, showing good agreement in both magnitude and temporal trends. Under air, the area‐equivalent diameter measured from cross‐sectional images increases from 0.34 to 2.75 µm over 6 h, whereas under oxygen it increases more gradually, reaching 1.04 µm. Figure 5d,e show rapid coalescence and densification under air, whereas Figure 5f,g retain finer microstructures under oxygen at comparable times. These results suggest that oxygen modifies the relative kinetics of competing processes during calcination rather than significantly altering the thermodynamic driving force for single‐crystal formation. Oxygen‐rich conditions are consistent with reduced effective grain‐boundary mobility and delayed coalescence, yielding smaller grain sizes at intermediate stages, whereas air accelerates coalescence and promotes larger domain formation. The agreement between simulations and experiments further supports the delayed emergence of single‐crystal domains under oxygen, and extends to an intermediate atmosphere of 0.06 MPa, where the measured size evolution is consistent with the prediction of the same parameter set (Figure S8). These microstructural differences are expected to influence electrochemical performance through their impact on grain size and domain development [40]. Such kinetic limitations under oxygen‐rich conditions may account for the limited electrochemical differences between samples calcined under single‐atmosphere conditions. Applying a single atmosphere throughout the calcination dwell for single‐crystal mid‐Ni cathode synthesis imposes an intrinsic trade‐off. Air enables rapid coalescence but allows unrestricted grain growth into broad size distributions (Figure 1c), whereas oxygen preserves uniformity but leaves coalescence incomplete within practical dwell times (Figure 5c). This trade‐off is difficult to resolve under a fixed atmosphere throughout the dwell, since rapid early‐stage coalescence and suppressed late‐stage growth favor different kinetic conditions. Calcination, however, proceeds through distinct stages, with coalescence dominating the early dwell and microstructure stabilization governing the later dwell, such that a kinetic condition favorable for one stage is not necessarily optimal for the other.

**FIGURE 5:**
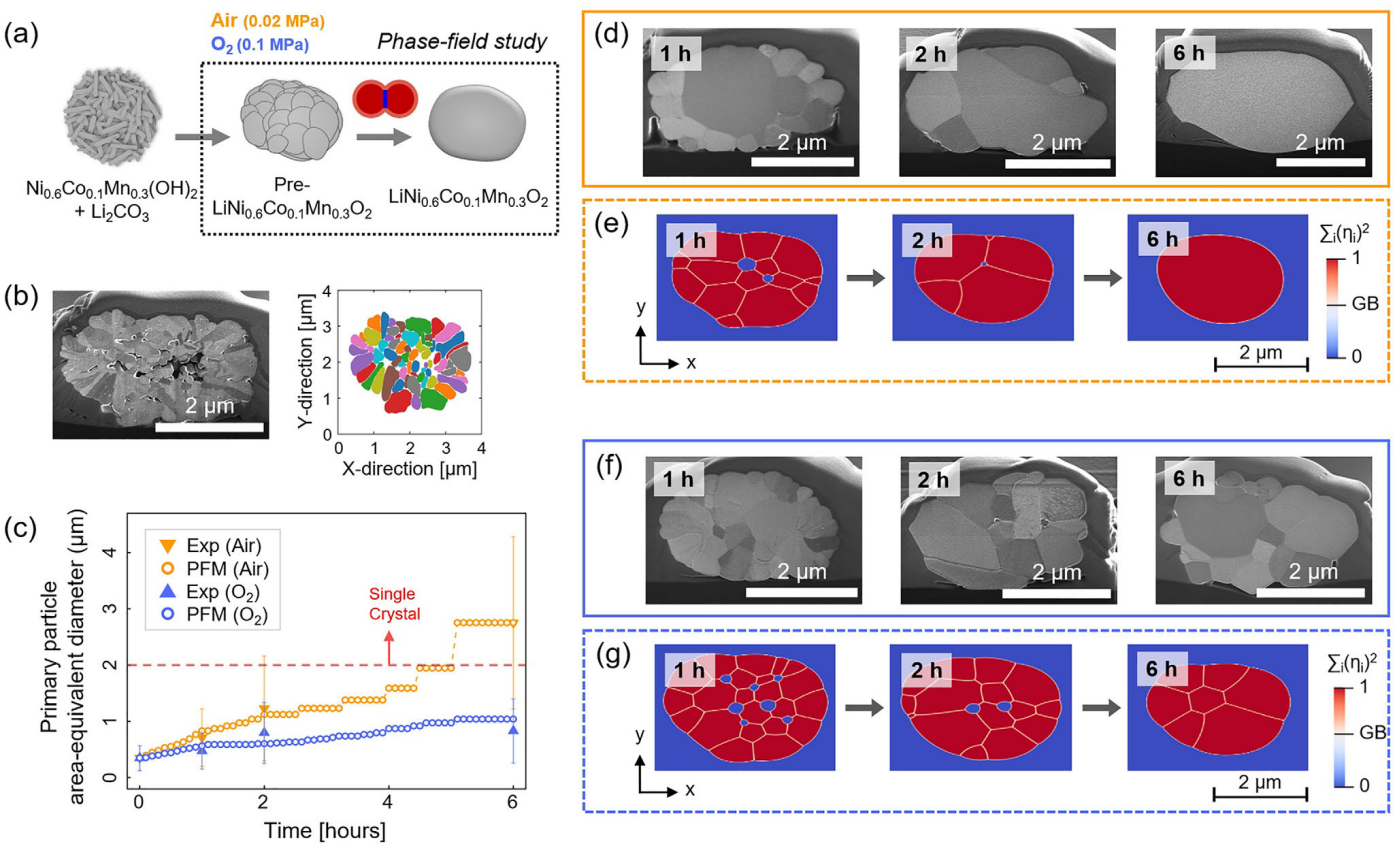
Experimental–computational comparison of oxygen‐controlled calcination kinetics. (a) Schematic illustration of morphological evolution. (b) Initial microstructure from SEM and digitized grain‐orientation field. (c) Area‐equivalent primary particle diameters from experiments and simulations. (d,e) Cross‐sectional SEM images and simulation results for air calcination. (f,g) Corresponding results for oxygen‐rich calcination.

### 2.5 Sequential Atmosphere Calcination and Electrochemical Performance

Stage‐specific oxygen control addresses this trade‐off by sequentially modulating grain‐boundary mobility across the calcination dwell. An initial air stage drives primary‐particle coalescence into single‐crystal domains, after which an oxygen stage suppresses further grain‐boundary migration to preserve the resulting microstructure. Because the four samples share an identical layered phase and composition, the observed differences in microstructure and cycling behavior can be attributed to the single‐crystal domain size and distribution established by sequential modulation, which can therefore be assessed directly through microstructural characterization and electrochemical cycling. The transition point, set at ∼4 h based on the simulated grain‐size evolution in Figure 5c and confirmed experimentally (Figure S9), is examined by comparing microstructure and cycling performance across four oxygen partial pressure profiles, namely air (6 h), O_2_ (6 h), air→O_2_, and O_2_→air.

Figure 6a shows representative surface and cross‐sectional SEM images for the two sequential conditions. The Air(4 h)→O_2_(2 h) sample exhibits well‐faceted, isolated single‐crystal particles with smooth surfaces and densified cross‐sections, consistent with substantial coalescence during the air stage followed by suppressed late‐stage grain growth under oxygen. In contrast, the O_2_(4 h)→Air(2 h) sample shows less regular particle morphology with internal pores visible in the cross‐sectional images. This is consistent with a kinetic sequence in which suppressed grain‐boundary mobility during the oxygen‐rich early stage delays primary‐particle merging, and the subsequent transition to air drives rapid late‐stage grain growth that encloses residual pores before they can escape to the surface. Figure 6b shows the primary particle thickness distributions for all four conditions. The Air(6 h) and Air→O_2_ samples show broad distributions with a tail extending beyond ∼2500 nm, indicative of unrestricted or late‐activated grain‐boundary migration. The O_2_(6 h) sample yields the narrowest distribution, centered at smaller mean thickness values, confirming that oxygen‐rich conditions suppress grain coarsening throughout the dwell. The O_2_→Air sample produces a distribution intermediate in breadth, with a mean shifted toward larger values than O_2_(6 h) while remaining substantially narrower than Air(6 h) or Air→O_2_. This is consistent with grain growth largely suppressed during the oxygen‐rich early stage and subsequently activated upon transition to air in the later dwell. These microstructural differences reflect the temporal sequencing of grain‐boundary mobility across the four processing routes and provide the structural basis for the electrochemical trends discussed below.

**FIGURE 6:**
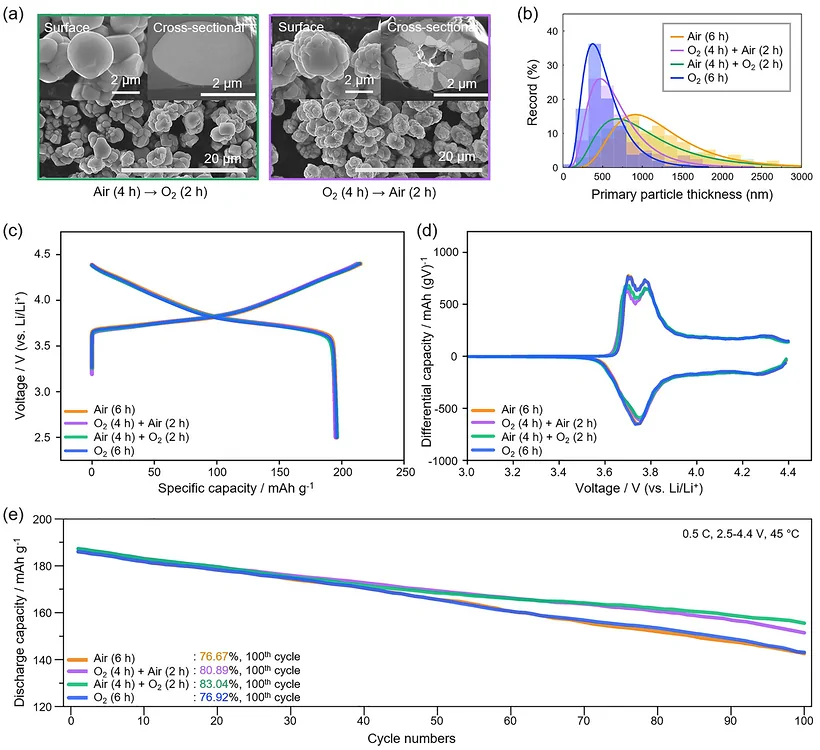
Microstructural and electrochemical characterization of SC‐NCM613 cathodes prepared under different oxygen partial pressure profiles during calcination. (a) Surface and cross‐sectional SEM images for Air(4 h)→O_2_(2 h) and O_2_(4 h)→Air(2 h) conditions. (b) Primary particle thickness distributions across all four calcination conditions. (c) Charge–discharge voltage profiles. (d) Differential capacity (dQ dV^−1^) curves. (e) Cycling performance over 100 cycles. Samples correspond to air, oxygen, and mixed‐atmosphere calcination conditions.

Electrochemical measurements in Figure 6c show comparable charge–discharge profiles across samples prepared under different oxygen partial pressure profiles, and the differential capacity curves in Figure 6d likewise show a consistent response across the four conditions. Cycling performance in Figure 6e shows that samples calcined exclusively under oxygen‐rich conditions, which retain polycrystalline structures within the limited dwell time of 6 h, exhibit capacity retention (76.92% after 100 cycles) comparable to those processed entirely in air (76.67%). This comparable retention indicates that, in this mid‐nickel system, oxygen‐rich conditions alone are insufficient to improve electrochemical performance when grain growth remains incomplete. The reduced grain‐boundary mobility under oxygen, while suppressing late‐stage coalescence, also limits the development of well‐formed single‐crystal domains within the same dwell time, leaving particle‐level microstructure dominated by polycrystalline character, undermining the durability advantages associated with single‐crystal morphology. Air calcination, in contrast, promotes coalescence to a similar overall extent but produces less uniform single‐crystal domains, since unrestricted grain‐boundary mobility throughout the dwell extends grain growth beyond the conditions favorable for homogeneous final microstructure. To address this trade‐off, the simulated grain‐size evolution in Figure 5c, which indicates substantial single‐crystal domain formation by ∼4 h of air calcination, was used to set the air‐stage duration in a two‐step protocol. The air–oxygen sequence first permits high grain‐boundary mobility under air, accelerating coalescence and single‐crystal domain formation; the subsequent transition to oxygen‐rich conditions suppresses further grain‐boundary migration, preserving the single‐crystal microstructure established during the air stage. Because the air–oxygen sequence and air‐only calcination share an identical initial air stage, the two processes diverge only in the later dwell. There, the transition to oxygen‐rich conditions selectively arrests the grain growth that would otherwise continue under sustained air, where it would extend single‐crystal domains beyond the range favorable for a uniform microstructure. In contrast, the reverse O_2_→air sequence suppresses coalescence at the early dwell when high grain‐boundary mobility is most needed, leaving the subsequent air stage to drive growth from a heterogeneous starting microstructure. As shown in Figure 6e, this sequential modulation approach yields improved capacity retention, reaching 83.04% after 100 cycles for the air–oxygen sequence, compared to 80.89% for the reverse sequence and ∼77% for single‐atmosphere processing. These results indicate that electrochemical performance is governed by temporal sequencing of grain‐boundary mobility, with the air–oxygen sequence achieving an optimal balance between early‐stage coalescence and late‐stage stabilization of the single‐crystal microstructure [41, 42].

## 3 Conclusion

In summary, this study establishes oxygen partial pressure as a key regulator of kinetic pathways during the calcination of mid‐nickel SC‐NCM cathodes. Combined experiments and phase‐field modeling identify grain‐boundary mobility as the dominant kinetic descriptor governing single‐crystal growth, with oxygen partial pressure modulating this parameter through changes in the apparent sintering activation barrier. While this treatment represents a simplified phenomenological description, it captures the primary trends in atmosphere‐dependent microstructural evolution and quantitatively reproduces experimentally observed mesoscale morphologies. Building on this framework, stage‐specific oxygen control addresses the intrinsic trade‐off of single‐atmosphere calcination by sequentially modulating grain‐boundary mobility across the calcination dwell, enabling time‐efficient single‐crystal formation with improved cycling stability. More broadly, this study demonstrates how kinetic descriptors extracted from mesoscale modeling can guide scalable SC cathode synthesis, providing mechanistic insight and a framework for predictive process design. Applying this framework to higher‐nickel compositions would require the atmosphere program to operate within a narrower window, because oxygen acts simultaneously as a kinetic variable and a phase stabilizer in these compositions, rather than as a kinetic variable alone. Identifying this window and recalibrating the oxygen‐dependent kinetic parameters accordingly remain the principal requirements for such an extension.

## 4 Experimental Section/Methods

### 4.1 Material Synthesis

Ni_0.6_Co_0.1_Mn_0.3_(OH)_2_ precursor was synthesized via a co‐precipitation method. A 4 L stirred reactor was used to maintain controlled reaction conditions (pH 11.0 ± 0.1, temperature 50°C ± 0.5°C, and stirring speed 1000 rpm). Stoichiometric amounts of NiSO_4_·6H_2_O, CoSO_4_·7H_2_O, and MnSO_4_·H_2_O were continuously fed into the reactor along with NaOH and NH_4_OH solutions as chelating agents. The precipitates were filtered, washed extensively with deionized water until reaching neutral pH, and dried under vacuum at 80°C for 12 h. The obtained precursor was mixed with stoichiometric Li_2_CO_3_ (Li/TM = 1.08:1) and calcined in a tube furnace at 950°C for 6 h with a heating rate of 5°C min^−1^ under a gas flow rate of 200 sccm. An oxygen atmosphere was maintained during the ramping process. During the holding step at 950°C, the gas atmosphere was controlled under the following conditions: O_2_ (6 h), O_2_ → air (4 h/2 h), air → O_2_ (4 h/2 h), and air (6 h). After calcination, the resulting powders were cooled to room temperature and stored in an argon‐filled glovebox to avoid moisture exposure.

### 4.2 Characterization

Crystallographic characterization was performed using X‐ray diffraction (XRD) with Cu Kα radiation (λ = 1.5406 Å) in a θ–2θ configuration on a Rigaku D/Max‐2500 V diffractometer. Diffraction patterns were collected over a 2θ range of 15°–120° with a step size of 0.01° and a scan speed of 2° min^−1^. The instrument was operated at 45 kV and 200 mA and equipped with a HyPix‐3000 detector. Rietveld refinements were performed based on the measured diffraction patterns. The crystal structure was refined using a layered hexagonal model (space group R3̅m) with Li/Ni cation mixing, allowing partial occupation of Ni in the Li layer and vice versa. The (003)/(104) ratio was evaluated from integrated peak intensities rather than peak heights. The morphologies and elemental compositions of the prepared cathodes were examined using field‐emission scanning electron microscopy (FE‐SEM, JEOL JSM‐7100F) equipped with energy‐dispersive X‐ray spectroscopy (EDS). Cross‐sectional microstructures of secondary particles were prepared using a focused ion beam (FIB, FEI Helios 650) after Pt deposition, followed by fine milling at ion beam currents of 2.5–0.79 nA to obtain clear grain boundary contrast. The bulk elemental composition of the calcined powders was determined by inductively coupled plasma optical emission spectroscopy (ICP‐OES).

### 4.3 Image Analysis

The thickness of primary particles was measured from surface SEM images, and the area‐equivalent diameter of primary particles was evaluated from FIB cross‐sectional images of internal microstructures using ImageJ. For the thickness measurements, more than 400 primary particles per calcination condition were analyzed from SEM images acquired at five or more regions of the sample. Pore fraction and particle size were determined from segmented images using custom Python routines. Binary masks were applied to distinguish pore and solid regions, and the pore fraction was calculated as the area fraction of pores within a defined region of interest. The area‐equivalent diameter of primary particles was obtained from the segmented particle areas. Image preprocessing included color‐based masking, region‐of‐interest extraction, and noise reduction to ensure consistent segmentation across datasets.

### 4.4 Electrochemical Analysis

SC‐NCM613 samples prepared under different gas atmosphere conditions were used as cathode materials. The half‐cell tests were performed through the following process. The powders were mixed with carbon black (Super P) and polyvinylidene fluoride (PVdF)‐based binder in a weight ratio of 90:6:4. *N*‐methyl‐2‐pyrrolidone (NMP) was added to adjust the rheological properties for optimal coating performance. The resulting slurry was uniformly coated onto aluminum foil using a doctor blade. The coated electrodes were dried at 120°C for 12 h under vacuum. The electrode loading levels were approximately 6.0 mg cm^−2^. The calendering process was precisely controlled using a gap‐controlled rolling press to achieve target electrode densities of 3.0–3.3 g cm^−3^, optimizing the balance between electronic conductivity and lithium‐ion diffusion pathways. Due to the inherent structural resilience of single‐crystalline materials, these particles exhibit elastic recovery post‐compression, so the calendering process must be performed immediately before cell assembly. The electrochemical performance of half cells was evaluated using 2032‐ type coin cells with lithium metal and 1 M LiPF_6_ in ethylene carbonate, ethyl methyl carbonate (3:7 vol%). Galvanostatic experiments of the samples were performed at 45°C with a constant current‐constant voltage (CC‐CV) charging protocol (CV step termination criterion: I < C/20). The formation process included a 10 h rest period followed by one cycle at C/10 rate and two cycles at C/5 rate. Subsequently, 100 cycles were conducted at C/2 rate to evaluate the cycle performance. All samples were cycled within a potential range of 2.5–4.4 V (vs. Li/Li^+^).

### 4.5 Phase‐Field Modeling

Details of the mesoscale‐level computational methodology are provided in the Supporting Information.

## Author Contributions

**Hyeonho Park**: conceptualization, methodology, software, data curation, investigation, validation, Writing – original draft, visualization. **Sushil Kumar**: software, data curation, validation, writing – original draft, visualization. **Sangbeom Kim**: methodology, writing – original draft, visualization. **Ho Choi**: project administration. **Youngmin Jeon**: project administration. **Taeju Bak**: project administration. **Jisook Hong**: project administration. **M. P. Gururajan**: writing – review and editing. **Youngjin Kim**: writing – review and editing, supervision, validation. **Kyoungdoc Kim**: conceptualization, project administration, supervision, funding acquisition, writing – review and editing, resources.

## Funding

This work was supported by POSCO Holdings and Korea Institute for Advancement of Technology (KIAT) funded by the Korea Government (MOTIE) (RS‐2024‐00419413, HRD Program for Industrial Innovation).

## Conflicts of Interest

The authors declare no conflicts of interest.

## Supporting information




**Supporting File**: advs78080‐sup‐0001‐SuppMat.docx.

## Data Availability

The data that support the findings of this study are available from the corresponding author upon reasonable request.
