# Supplementary material for: Oxygen Partial Pressure as a Kinetic Descriptor for Single‐Crystal Morphology in Mid‐Nickel Layered Cathodes
Source: Adv Sci (Weinh). 2026 Sep 29:e78080. Online ahead of print. doi: 10.1002/advs.78080 (PMC13624330; doi:10.1002/advs.78080)
Supplement: Supplementary file 1 [file ADVS-9999-e78080-s001.docx]

Supporting Information

Oxygen Partial Pressure as a Kinetic Descriptor for Single-Crystal Morphology in Mid-Nickel Layered Cathodes

*Hyeonho Park, Sushil Kumar, Sangbeom Kim, Ho Choi, Youngmin Jeon, Taeju Bak, Jisook Hong, M. P. Gururajan, Youngjin Kim,* and Kyoungdoc Kim**

H. Park, S. Kumar, Prof. K. Kim

Graduate Institute of Ferrous and Eco Materials Technology, Pohang University of Science and Technology, Pohang 37673, Republic of Korea

S. Kim, Prof. Y. Kim

Department of Battery Convergence Engineering, Kangwon National University, Chuncheon 24341, Republic of Korea

Dr. H. Choi, Dr. Y. Jeon, Dr. T. Bak, Dr. J. Hong

AI & Robotics Convergence Lab, POSCO N.EX.T Hub, Seoul 06194, Republic of Korea

Prof. M. P. Gururajan

Department of Metallurgical Engineering and Materials Science, IIT Bombay, Powai Mumbai, Maharashtra 400076, India

Email: kdkim@postech.ac.kr (K. Kim), ykim@kangwon.ac.kr (Y. Kim)

Hyeonho Park, Sushil Kumar and Sangbeom Kim contributed equally to this work.

**Figure S1.** SEM images of Ni_0.6_Co_0.1_Mn_0.3_(OH)_2_ precursors.

**Figure S2.** Rietveld-refined X-ray diffraction (XRD) patterns of NCM613 calcined at 950 °C under different atmospheres and dwell times. (a-c) held in O_2_ for 0, 2, and 6 h, respectively; (d, e) switched to air and held for 2 and 6 h.

**Figure S3.** SEM images showing the EDS line-scan positions and corresponding Ni, Co, and Mn profiles for NCM613. (a) At the beginning of the dwell. (b, c) After 6 h at p(O_2_) = 0.06 and 0.10 MPa, respectively. The corresponding data for air (0.02 MPa) are shown in Figure 1f.

**Figure S4.** Early-stage densification behavior from phase-field simulations under idealized configurations for simulation times of τ ≤ 1000.

**Figure S5.** Microstructural features formed under rapid heating in air. (a) Cross-sectional SEM image of a secondary particle calcined at a heating rate of 10 °C min^-1^, showing a hollow structure with a large central pore. (b) Corresponding pore fraction for samples processed in air (10 °C min^-1^) and O_2_ (5 °C min^-1^).

**Figure S6.** Calcination behavior of a precursor composed of loosely aggregated primary particles. (a) SEM images of Ni_0.6_Co_0.1_Mn_0.3_(OH)_2_ precursors with morphologies distinct from those used in this study. (b) Cross-sectional SEM image of a secondary particle at 950 °C under oxygen flow.

**Figure S7.** Phase-field simulations at p(O_2_) = 0.02, 0.04, 0.06, 0.08, and 0.10 MPa at 950 °C. (a) Activation energy and (b) effective grain-boundary mobility during the calcination dwell. Insets show magnified views of the 0.08 and 0.10 MPa curves.

**Figure S8.** NCM613 calcined at 950 °C under a premixed O_2_–N_2_ atmosphere at p(O_2_) = 0.06 MPa for 1, 2, and 6 h. (a) Surface SEM images. (b) Cross-sectional SEM images and (c) the corresponding phase-field simulations. (d) Experimental and simulated area-equivalent primary-particle diameters, compared with the air and oxygen data from Figure 5c.

**Figure S9.** NCM613 recovered after 4 h under air, at the transition point of the air→O_2_ protocol. (a) Surface SEM image and (b) cross-sectional SEM image after FIB milling.

**Phase-field modeling**

***Theoretical Methods***

A phase-field framework was employed to describe microstructural evolution during the calcination of mid-nickel LiNi_0.6_Co_0.1_Mn_0.3_O_2_ (NCM613) toward single-crystal formation. At elevated temperatures, precursor particles undergo concurrent chemical reactions and sintering processes, including densification and grain growth, which are intrinsically coupled through diffusion and interfacial energetics. The microstructure is described by conserved and non-conserved field variables representing material distribution and grain structure, respectively. These fields evolve according to free-energy-based partial differential equations, enabling a unified treatment of mass transport and interfacial migration. Densification arises from diffusion-mediated material redistribution, whereas grain growth is governed by curvature-driven interface motion. This framework enables quantitative analysis of the competition between densification and grain coarsening, which governs the emergence of single-crystal morphology in NCM613 during calcination.

***Sintering***

Sintering involves the evolution of mass density, particle morphology, spatial arrangement, and crystallographic orientation. The phase-field formulation couples the mass density field with a multicomponent structural order parameter field, yielding a total free energy, *F*, expressed as a functional of ρ and η.

$F=\int\left[ f\left( \rho,\eta_{\alpha} \right)+\frac{1}{2}\beta_{\rho}|\vec{\nabla}\rho|^{2}+\sum_{\alpha} \frac{1}{2}\beta_{\eta}\left| \vec{\nabla}\eta_{\alpha} \right|^{2} \right]dv$	(S1)

The first term represents the bulk free energy density governing the coexistence of solid and pore phases. The second and third terms correspond to surface and grain-boundary energy contributions, respectively. The bulk free energy term is defined as:

$f\left( \rho,\eta_{\alpha} \right)=A\rho^{2}(1-\rho)^{2}+B\left[ \rho^{2}+6(1-\rho)\sum_{\alpha} \eta_{\alpha}^{2}-4(2-\rho)\sum_{\alpha} \eta_{\alpha}^{3}+3\left( \sum_{\alpha} \eta_{\alpha}^{2} \right)^{2} \right]$	(S2)

Here, A and B are constants whose magnitudes depend on the grain-boundary energy (γ_gb_) and surface energy (γ_surf_) of the material. The parameters β_ρ_, β_η_, A, and B can be expressed in terms of γ_gb_, γ_surf_, and the interfacial width (*l*) as given below.

$A=\frac{12\gamma_{surf}-7\gamma_{gb}}{l}$	(S3)

$B=\frac{\gamma_{gb}}{l}$	(S4)

$\beta_{\rho}=\frac{3l}{4}(2\gamma_{surf}-\gamma_{gb})$	(S5)

$\beta_{\eta}=\frac{3}{4}\gamma_{gb}l$	(S6)

The mass density field satisfies ρ = 0 in pores and ρ = 1 in the solid phase. The multicomponent order parameter η_α_ is zero in pores and takes unit vector values to represent different grain orientations. During sintering, ρ and η evolve concurrently to describe microstructural evolution. The evolution of ρ is governed by the Cahn–Hilliard equation:

$\frac{\partial\rho}{\partial t}=\vec{\nabla}\cdot\left( D\vec{\nabla}\frac{\delta F}{\delta\rho}-\rho\vec{v}_{\mathrm{adv}} \right)$	(S7)

Here, *D* denotes the diffusion coefficient, which is associated with diffusivity in bulk (*D*_vol_), surface (*D*_surf_), and gain boundary (*D*_gb_).

$D=D_{vol}\phi(\rho)+D_{surf}\rho^{2}\left( 1-\rho^{2} \right)+D_{\mathrm{gb}}\sum_{\alpha} \sum_{\alpha^{'}\neq\alpha} \eta_{\alpha}\eta_{\alpha^{'}}$	(S8)

where $\phi(\rho)=\rho^{3}\left( 10-15\rho+6\rho^{2} \right)$

The non-conserved order parameter, η, describes the grain structure, and grain-boundary migration is governed by the Allen–Cahn equation:

$\frac{\partial\eta_{\alpha}}{\partial t}=-L\frac{\delta F}{\delta\eta_{\alpha}}-\vec{\nabla}\cdot\left[ \eta_{\alpha}\vec{v}_{\mathrm{adv}} \right]$	(S9)

where L is mobility of grain boundary migration. The advection flux describes mass transport due to the motion of local material elements as rigid bodies. This rigid-body motion consists of translational and rotational components of the velocity field.

$\vec{v}_{\mathrm{adv}}=\sum_{\alpha} \vec{v}_{\mathrm{adv}}(\alpha)=\sum_{\alpha} \left[ \vec{v}_{t}(\alpha)+\vec{v}_{r}(\alpha) \right]$	(S10)

The effective local force driving particles toward each other arises from rigid-body translation and rotational motion. This force is defined as:

$d\vec{F}(\alpha)=\kappa\sum_{\alpha^{'}\neq\alpha} \left( \rho-\rho_{0} \right)\left\langle\eta_{\alpha}\eta_{\alpha^{'}} \right\rangle\left[ \nabla\eta_{\alpha}-\nabla\eta_{\alpha^{'}} \right]d^{3}r$	(S11)

$\left\langle\eta_{\alpha}\eta_{\alpha^{'}} \right\rangle=\left\{ \begin{matrix} 1, & \text{ if }\eta_{\alpha}\eta_{\alpha^{'}}\geq c \\ 0, & \text{ otherwise } \end{matrix} \right.$	(S12)

where κ is a stiffness constant that relates the force magnitude to vacancy supersaturation at grain boundaries. The parameter $\rho_{0}$ denotes the equilibrium mass density at grain boundaries. The product $\left\langle\eta_{\alpha}\eta_{\alpha^{'}} \right\rangle$ identifies grain boundaries when it exceeds a critical value, c, and the gradient difference $\left[ \nabla\eta_{\alpha}-\nabla\eta_{\alpha^{'}} \right]$ defines the interaction force between neighboring grains. The resultant force and torque acting on the αth particle about its center of mass are:

$\vec{F}(\alpha)=\int_{V} d\vec{F}(\alpha)$	(S13)

$\vec{T}(\alpha)=\int_{V} \left[ r-r_{c}(\alpha) \right]\times d\vec{T}(\alpha)$	(S14)

The center of mass and volume of particles are defined as:

$r_{c}(\alpha)=\frac{1}{V(\alpha)}\int_{V} r\eta(r,\alpha)d^{3}r$	(S15)

The velocity fields of rigid-body translation and rotation of particles are:

$\vec{v}_{t}(r,\alpha)=\frac{m_{t}}{V(\alpha)}\vec{F}(\alpha)\eta(r,\alpha)$	(S16)

$\vec{v}_{r}(r,\alpha)=\frac{m_{r}}{V(\alpha)}\vec{T}(\alpha)\times\left[ r-r_{c}(\alpha) \right]\eta(r,\alpha)$	(S17)

where m_t_ and m_r_ are translational and rotational mobilities.

***Oxygen Diffusion***

The oxygen transport inside NCM613 particles during calcination was described using a Cahn–Hilliard diffusion equation:

$\frac{\partial\tilde{c}_{O_{2}}}{\partial t}=\vec{\nabla}\cdot\left( D_{O_{2}}\vec{\nabla}\mu_{O_{2}} \right)-\vec{\nabla}\cdot\left( \tilde{c}_{O_{2}}\vec{v} \right)+R_{O_{2}}$	(S18)

where the chemical potential is defined as

$\mu_{O_{2}}=\delta F_{O_{2}}/\delta\tilde{c}_{O_{2}}$	(S19)

and the free-energy functional is given by

$F_{O_{2}}=\int\left\{ f\left( \tilde{c}_{O_{2}} \right)+\frac{1}{2}\kappa_{O_{2}}\left| \nabla\tilde{c}_{O_{2}} \right|^{2} \right\}dv$	(S20)

The bulk free-energy density was defined using a double-well potential:

$f\left( \tilde{c}_{O_{2}} \right)=A_{O_{2}}\left( \tilde{c}_{O_{2}}-c_{g,O_{2}} \right)^{2}\left( \tilde{c}_{O_{2}}-c_{p,O_{2}} \right)^{2}$	(S21)

The model consists of a two-phase system comprising a surrounding gas matrix and an NCM particle phase. The equilibrium oxygen concentrations in these phases are denoted by $c_{g,O_{2}}$ and $c_{p,O_{2}}$, respectively, with $c_{g,O_{2}}=1$. The particle-phase equilibrium concentration, $c_{p,O_{2}}$, is determined by the ambient oxygen partial pressure through the defect-equilibrium relation described below. The equilibrium oxygen concentration inside the particle depends on the ambient oxygen partial pressure and is determined using the defect-equilibrium relation discussed in the following section. The gradient-energy coefficient and bulk-energy coefficient are defined as:

$A_{O_{2}}=\frac{12\gamma_{O_{2}}}{l}$	(S22)

$\kappa_{O_{2}}=\frac{3}{2}\gamma_{O_{2}}l$	(S23)

where $\gamma_{O_{2}}$ is the oxygen-interface energy and $l$ is the diffuse-interface width. During calcination, oxygen exchange between the surrounding atmosphere and the particle surface is modeled using the reaction term:

$R_{O_{2}}=\phi\left( \rho\right)\cdot k_{reac,O_{2}}\cdot\left( c_{p,O_{2}}-\tilde{c}_{O_{2}} \right)$	(S24)

where $\phi\left( \rho\right)$ is an interpolation function localizing the reaction near the particle interface. The reaction-rate coefficient is expressed using an Arrhenius relation:

$k_{\text{reac},O_{2}}=k_{0,\text{reac},O_{2}}\exp\left( \frac{-E_{\text{reac },O_{2}}}{RT} \right)$	(S25)

where $k_{0,\text{reac},O_{2}}$ is the pre-exponential reaction constant, $E_{\text{reac },O_{2}}$ is the activation energy for oxygen incorporation, *R* is the universal gas constant, and *T* is the absolute temperature. The oxidation reaction associated with NCM calcination under different oxygen partial-pressure conditions can be written as

$\frac{1}{2}O_{2}\left( g \right)+V_{O}^{\bullet\bullet}\to O_{O}^{x}+2h^{\bullet}$	(S26)

where $V_{O}^{\bullet\bullet}$ represents an oxygen vacancy, $O_{O}^{x}$ denotes an oxygen ion occupying a regular lattice site, and $h^{\bullet}$ represents an electron hole. This reaction describes the incorporation of oxygen from the gas phase into oxygen-vacancy sites within the NCM lattice. The annihilation of oxygen vacancies is charge compensated by the formation of electron holes in the valence band.

The corresponding mass-action relation is given by:

$K=\frac{\left[ O_{O}^{x} \right]\left[ h^{\bullet} \right]^{2}}{\left[ V_{O}^{\bullet\bullet} \right]\left( p_{O_{2}} \right)^{1/2}}$	(S27)

The oxygen-site conservation relation can be written as

$\left[ O_{O}^{x} \right]+\left[ V_{O}^{\bullet\bullet} \right]=1$	(S28)

The equilibrium oxygen concentration within the NCM particle is defined as

$c_{O_{2}}^{\mathrm{eq}}=\left[ O_{O}^{x} \right]$	(S29)

$c_{O_{2}}^{\mathrm{eq}}=1-\left[ V_{O}^{\bullet\bullet} \right]$	(S30)

Rearranging the mass-action expression gives

$\left[ V_{O}^{\bullet\bullet} \right]=\frac{\left[ h^{\bullet} \right]^{2}}{K\left( p_{O_{2}} \right)^{1/2}}$	(S31)

Substituting this relation into the oxygen-site conservation equation yields

$c_{O_{2}}^{\mathrm{eq}}=1-\frac{\left[ h^{\bullet} \right]^{2}}{K\left( p_{O_{2}} \right)^{1/2}}$	(S32)

By defining the oxygen-vacancy equilibrium parameter as

$K_{v}=\frac{\left[ h^{\bullet} \right]^{2}}{K}$	(S33)

the equilibrium oxygen concentration becomes

$c_{O_{2}}^{\mathrm{eq}}=1-K_{v}\left( p_{O_{2}} \right)^{-1/2}$	(S34)

This relation indicates that increasing oxygen partial pressure reduces the oxygen vacancy concentration and increases the equilibrium oxygen occupancy within the NCM lattice. The study was performed under two different oxygen partial-pressure conditions: 0.02 MPa and 0.1 MPa. The oxygen partial pressure was nondimensionalized as:

$p_{O_{2}}^{*}=\frac{p_{O_{2}}}{p_{\mathrm{ref}}}$	(S35)

where the reference oxygen partial pressure is taken as $p_{\mathrm{ref}}=$ 0.02 MPa and the oxygen-vacancy equilibrium parameter is chosen as $K_{v}=$ 0.45. Using the equilibrium relation:

$c_{O_{2}}^{\mathrm{eq}}=1-K_{v}\left( p_{O_{2}}^{*} \right)^{-1/2}$	(S36)

the equilibrium oxygen concentrations inside the particle are obtained as

$c_{O_{2}}^{\mathrm{eq}}=0.55, p_{O_{2}}=0.02 \mathrm{Mpa}$

$c_{O_{2}}^{\mathrm{eq}}=0.80, p_{O_{2}}=0.1 \mathrm{Mpa}$

***Lithium diffusion***

Lithium transport inside the NCM613 particles during calcination was modeled using Fick’s second law of diffusion:

$\frac{\partial\tilde{c}_{\mathrm{Li}}}{\partial t}=\vec{\nabla}\cdot\left( D_{\mathrm{Li}}\vec{\nabla}\tilde{c}_{\mathrm{Li}} \right)-\vec{\nabla}\cdot\left( \tilde{c}_{\mathrm{Li}}\vec{v} \right)+R_{\mathrm{Li}}$	(S37)

where $\tilde{c}_{\mathrm{Li}}$ is the lithium concentration, $D_{\mathrm{Li}}$ is the lithium diffusivity, $\vec{v}$ is the velocity field associated with microstructural evolution, and $R_{\mathrm{Li}}$ is the lithium reaction term. Fick’s second law was employed for lithium transport because lithium diffusion during calcination is primarily governed by concentration-gradient-driven solid-state diffusion within the particle. Unlike oxygen transport, which involves defect-mediated thermodynamic equilibrium with the surrounding atmosphere, lithium incorporation is mainly controlled by bulk diffusion from the lithium source into the evolving NCM particle. Therefore, a concentration-based diffusion model is sufficient to describe lithium redistribution during calcination. Li diffuses from the lithium source toward the NCM particles during calcination and reacts at the particle surface. The interfacial lithium incorporation reaction is modeled using the source term:

$R_{\mathrm{Li}}=\phi(\rho)\cdot k_{\text{reac},Li}\cdot\left( 1-\tilde{c}_{\mathrm{Li}} \right)\cdot\tilde{c}_{O_{2}}$	(S38)

where $\phi\left( \rho\right)$ localizes the reaction near the particle interface and $k_{r,Li}$ is the lithium reaction-rate coefficient. The factor $\left( 1-c_{\mathrm{Li}} \right)$ represents the availability of vacant lithium sites inside the particle, while $c_{O_{2}}$ accounts for the influence of local oxygen concentration on lithium incorporation. The dependence on oxygen concentration reflects the coupled nature of lithium incorporation and oxygen occupancy during NCM calcination. Higher local oxygen concentration promotes lithium incorporation by stabilizing the layered oxide structure and reducing oxygen-deficient states. The lithium reaction-rate coefficient is described using an Arrhenius relation:

$k_{\text{reac },Li}=k_{0,\text{ reac },Li}exp\left( \frac{-E_{\text{reac },Li}}{RT} \right)$	(S39)

where $k_{0,Li}$ is the pre-exponential reaction constant, $E_{r,Li}$ is the activation energy for lithium incorporation. The reaction term $R_{\mathrm{Li}}$ acts as a source term in the diffusion equation and drives lithium incorporation into the NCM particles during calcination. Surface reactions are modeled using a concentration-dependent rate expression, in which the reaction rate is coupled to the local oxygen concentration, reflecting the oxygen-dependent nature of lithiation.

$D=D_{0}\exp\left( \frac{-E_{a}}{R_{g}T} \right)$	(S40)

$D_{O_{2}}=D_{0,\text{diff,}O_{2}}\exp\left( \frac{-Q_{v,O_{2}}}{RT} \right)$	(S41)

$D_{\mathrm{Li}}=D_{0,diff,Li}exp\left( \frac{-Q_{v,Li}}{RT} \right)$	(S42)

$L=\frac{4D_{gb,0}}{3l}\exp\left( \frac{-E_{a}}{R_{g}T} \right)\cdot\frac{\Omega}{kT}$	(S43)

All transport coefficients are described using Arrhenius-type relations, with distinct diffusion coefficients assigned to bulk, surface, and grain-boundary transport. A spatially uniform temperature is assumed owing to the small computational domain. Grain-boundary mobility is linked to diffusivity through a relation involving the atomic volume, Ω, and the Boltzmann constant, k, assuming diffusion-controlled migration.

**Table S1.** Summary of parameters used for phase-field model.

| Variable name | Symbol | Value | Unit | Reference |
| --- | --- | --- | --- | --- |
| Interfacial width | *w* | 30.0 | nm | Assumed |
| Activation energy for calcination | *E_a,_*_0_ | 50.0 | kJ/mol | [2] |
| Extra activation energy for lithiated particles | *E_a,_*_Li_ | 80.0 | kJ/mol | [2] |
| Extra activation energy for particles with adsorbed oxygen | *E_a,_*_O2_ | 30.0 | kJ/mol | [2] |
| Surface energy for LiNi_x_Mn_y_Co_1-x-y_O_2_ | *γ_surf_* | 1.0 | J/m^2^ | [4] |
| Grain-boundary energy for LiNi_x_Mn_y_Co_1-x-y_O_2_ | *γ_gb_* | 0.42 | J/m^2^ | [2] |
| Pre-exponential factor for the bulk diffusion | *D_vol_* | 1.16x10^-18^ | m^2^/s | [3] |
| Pre-exponential factor for the surface diffusion | *D_surf_* | 4.64x10^-16^ | m^2^/s | [3] |
| Pre-exponential factor for the grain-boundary diffusion | *D_gb_* | 4.64x10^-17^ | m^2^/s | [3] |
| Activation energy for lithium diffusion | *Q*_v,Li_ | 30.0 | kJ/mol | [2] |
| Pre-exponential factor for lithium diffusion | *D*_0,diff,Li_ | 1.50x10^-18^ | m^2^/s | [2] |
| Activation energy for lithium reaction | *E*_reac,Li_ | 20.0 | kJ/mol | [3] |
| Activation energy for oxygen diffusion | *Q*_v,O2_ | 10.0 | kJ/mol | [5] |
| Pre-exponential factor for oxygen diffusion | *D*_0,diff,O2_ | 1.0x10^-17^ | m^2^/s | [5] |
| Activation energy for oxygen reaction | *E*_reac,O2_ | 10.0 | kJ/mol | [2] |
| Reaction rate for oxidation at the particle surface | $k_{\text{reac},O_{2}}$ | 1/s | $k_{\text{reac},Li}/3$ | [3] |

**Table S2.** Elemental compositions of NCM613 determined by ICP-OES.

| Condition | Li (wt%) | Ni (wt%) | Co (wt%) | Mn (wt%) | Li/(Ni+Co+Mn) | Composition |
| --- | --- | --- | --- | --- | --- | --- |
| O_2_, 0 h | 7.71 | 35.63 | 6.19 | 16.68 | 1.09 | Ni_0.60_Co_0.10_Mn_0.30_ |
| Air, 6 h | 7.21 | 36.42 | 6.33 | 17.17 | 1.00 | Ni_0.60_Co_0.10_Mn_0.30_ |
| O_2_, 6 h | 7.47 | 35.55 | 6.24 | 16.72 | 1.06 | Ni_0.60_Co_0.10_Mn_0.30_ |

***Non-dimensionalization of Parameters***

The parameters used in the simulation are listed in the table. For numerical implementation, all physical parameters are converted into nondimensional form using the following relations:

Length (*l*): $l^{*}=\frac{l}{l_{0}}$

Activation Energy (*E_a_*): $E_{a}^{*}=\frac{E_{a}}{RT}$

Diffusivity pre-exponential factor (*D_0_*): $D_{0}^{*}=\frac{D_{0}}{D_{ref}}$

Time (*t*): $t^{*}=\frac{tD_{ref}}{l_{0}^{2}}$

Surface Energy (*γ*): $\gamma^{*}=\frac{\gamma V_{m}}{RTl_{0}}$

Energy density coefficient (*A*): $A^{*}=\frac{AV_{m}}{RT}$

Gradient coefficient (*κ*): $\kappa^{*}=\frac{\kappa V_{m}}{RTl_{0}^{2}}$

The superscript ∗ denotes the nondimensional quantity. Based on the interface width (*w*), the characteristic length scale: *l_0_* = 12 nm, the reference diffusivity: *D_ref_* = 10^-18^ m^2^/s, and the molar volume: *V_m_* = 9.44×10^-6^ m^3^/mol, respectively.

**References**

[1] Y. U. Wang, *Acta Mater.* **2006**, *54*, 953–961.
[2] M. Wolfman, X. Wang, J. C. Garcia, P. Barai, J. E. Stubbs, P. J. Eng, O. Kahvecioglu, T. L. Kinnibrugh, K. E. Madsen, H. Iddir, V. Srinivasan, T. T. Fister, *Adv. Energy Mater.* **2022**, *12*, 2102951.
[3] A. Tayal, P. Barai, H. Zhong, O. Kahvecioglu, X. Wang, K. Z. Pupek, L. Ma, S. N. Ehrlich, V. Srinivasan, X. Qu, J. Bai, F. Wang, *Adv. Mater.* **2024**, *36*, 2312027.
[4] J. C. Garcia, J. Bareño, J. Yan, G. Chen, A. Hauser, J. R. Croy, H. Iddir, *J. Phys. Chem. C* **2017**, *121*, 8290–8299.
[5] A. Ghosh, J. M. Foster, G. Offer, M. Marinescu, *J. Electrochem. Soc.* **2021**, *168*, 020509.
[6] S.-J. L. Kang, *Sintering: Densification, Grain Growth, and Microstructure*, Elsevier, Amsterdam **2004**.
[7] D. Turnbull, *J. Met.* **1951**, *3*, 661–665.
[8] M. N. Rahaman, *Sintering of Ceramics*, CRC Press, Boca Raton, FL **2008**.
